# Treatment of Brain AVMs (TOBAS): study protocol for a pragmatic randomized controlled trial

**DOI:** 10.1186/s13063-015-1019-0

**Published:** 2015-11-04

**Authors:** Tim E. Darsaut, Elsa Magro, Jean-Christophe Gentric, André Lima Batista, Chiraz Chaalala, David Roberge, Michel W. Bojanowski, Alain Weill, Daniel Roy, Jean Raymond

**Affiliations:** Department of Surgery, Division of Neurosurgery, University of Alberta Hospital, Mackenzie Health Sciences Center, Edmonton, AB Canada; Department of Surgery, Service of Neurosurgery, Centre Hospitalier de l’Université de Montréal (CHUM), Notre-Dame Hospital, Montreal, QC Canada; Service de Neurochirurgie, CHU Cavale Blanche, INSERM UMR 1101 LaTIM, Brest, France; Department of Radiology, Service of Neuroradiology, Centre Hospitalier de l’Université de Montréal (CHUM), Notre-Dame Hospital, Interventional Neuroradiology (NRI), 1560 Sherbrooke East, Pavillion Simard, room Z12909, Montreal, QC H2L 4 M1 Canada; Service de Radiologie, CHU Cavale Blanche, EA 3878 GETBO Brest, France; Department of Radio-Oncology, Centre Hospitalier de l’Université de Montréal (CHUM), Notre-Dame Hospital, Montreal, QC Canada

**Keywords:** Brain arteriovenous malformation, Randomized trial, Care trial, Ruptured AVM, Unruptured AVM, Embolization, Radiosurgery, Neurosurgery

## Abstract

**Background:**

The management of unruptured brain arteriovenous malformation (AVM) patients remains controversial. Furthermore, curative attempts to treat *ruptured* AVM patients have not been questioned so far, and there is a lack of prospective data on clinical results according to treatment modality. Endovascular treatment is often used aiming to improve the safety or efficacy of surgery or radiation therapy, but benefits have never been documented in a trial. A care trial context is needed to evaluate interventions at the same time they are practised.

**Methods/Trial design:**

TOBAS is a pragmatic, prospective, multicenter, randomized, controlled trial and registry which offers a care trial context for brain AVM patients, including surgical resection, radiosurgery or endovascular embolization, alone or combined. The study includes two RCTs and a multimodality prospective registry. The objectives of the proposed study are to assess whether preventive interventions (surgery, embolization, radiation therapy, alone or combined), selected by the local treatment team and performed as locally practiced, randomly allocated and compared with conservative management, in unruptured brain AVM patients eligible for active or conservative management, can improve the proportion of patients having an independent outcome (modified Rankin Scale (mRS) < 3, as assessed by a standardized questionnaire administered by non-masked care personnel) at 10 years. All patients judged ineligible for randomized allocation are to be entered in a multimodal registry. The objective of a nested trial in patients with ruptured or unruptured AVMs to be treated by surgery or radiation therapy, is to assess whether pre-surgical or pre-radiation embolization, randomly allocated and compared with no embolization, can improve the proportion of patients with complete eradication of the AVM, as locally adjudicated, combined with a good clinical outcome (mRS < 3). The study will require up to 2000 patients in approximately 30 centers or more, followed for 10 years. TOBAS is registered at clinicaltrials.gov: NCT02098252 as of 25 March 2014.

**Electronic supplementary material:**

The online version of this article (doi:10.1186/s13063-015-1019-0) contains supplementary material, which is available to authorized users.

## Background

Cerebral arteriovenous malformations (AVMs) are complex, heterogeneous, uncommon lesions that can lead to significant neurological disability or death, most commonly from intracranial hemorrhage (ICH) [1, 2]. Intracranial AVMs are typically diagnosed before the age of 40, approximately 40–50 % with a hemorrhagic presentation. An AVM-related seizure is reported as the presenting feature in 20–35 % of cases and although these can be successfully managed with anti-epileptic agents, some AVMs lead to intractable seizures in spite of medication. Other presentations include headaches or focal neurological deficits. AVMs can also be incidental findings during investigation of unrelated symptoms. Population-based data suggest that the annual incidence of discovery of a symptomatic AVM is approximately 1 per 100,000 population [1–5].

The management of brain AVMs is controversial and complex. Three different modalities are currently used (surgical resection; endovascular embolization; radiotherapy) alone or in various combinations, depending on local expertise, size, location of the lesion, and clinical presentation. Over the last decade, there has been an evolution of microsurgical as well as endovascular and radiosurgical techniques to treat these lesions [6–13]. As the management options have evolved, individual and combined modality treatment protocols have developed in various directions in different institutions. The choice of management is largely dependent on the decisions of the local physicians that make up the treatment team, and a recent survey has demonstrated substantial variability in decision-making for almost all types of AVMs [14]. Interventional therapies, when they are performed, are assumed to decrease the risk of initial or subsequent hemorrhage and, therefore, lead to better long-term outcomes, an assumption that has yet to be proven, since none of the treatment modalities or combinations thereof has been shown to improve outcomes for brain AVM patients. While microsurgical removal may provide a relatively safe and immediate cure for superficial AVMs in non-eloquent brain, resection of malformations in certain locations with a large nidus, deep draining veins, or high-flow shunts may carry a relatively high risk of morbidity [11, 13, 15–17]. Embolization can be performed with curative intent for some AVMs with favorable angio-architecture. Recently, embolization with Onyx has increasingly been proposed as a primary curative procedure [18–20]. This treatment modality is also performed as an adjunct to other modalities, either to render surgery technically simpler and possibly with less morbidity, or to reduce the AVM size to make it more conducive to cure with radiation therapy. The potential benefits of pre-surgical embolization have never been proven. Therapy may be initiated with a number of embolization sessions, typically over a span of several months. When endovascular treatment sessions have not successfully obliterated the AVM, treatment may be completed by radiation therapy, which takes 2–3 years to occlude the AVM in up to 80 % of cases if the nidus has been rendered relatively small (2 cm or less) [10]. As with embolization, radiation therapy can also be performed primarily with curative intent, particularly for small, surgically inaccessible or less safely accessible lesions. The merits of endovascular treatment prior to radiation therapy have been questioned, as the presence of endovascular material has been purported to hinder the obliteration of AVMs treated with radiation therapy, although this too remains contentious [21–23].

Irrespective of initial treatment modality chosen, therapy is sometimes suspended or interrupted because of a complication (transient or permanent). The benefits of pre-surgical or pre-radiation therapy embolization have recently been questioned [10, 24]. Although the question of which AVM treatment modality is the most appropriate first choice remains controversial, consensus can be reached in some circumstances. Surgical evacuation of a life-threatening hematoma exerting significant mass effect may remain uncontested, even though many patients with a hemorrhagic presentation do not necessarily meet this threshold for surgical intervention. Almost all other management choices remain debatable. A systematic review has proposed that approximately 7.1 % of surgical candidates, 6.6 % of endovascular candidates, and 5.1 % of radiosurgical candidates experience permanent neurological deficits after treatment, with outcomes certainly influenced by case selection [13]. The epidemiological study of Davies et al., using the Nationwide Inpatient Sample (NIS) data base and surrogates such as location at discharge, compared outcomes following AVM treatment with the different modalities, and found the worst outcomes were obtained when endovascular treatment was combined with surgical management, for both ruptured and unruptured AVMs [7]. All current therapeutic options involve substantial risks of mortality and morbidity (ranging from 1–30 %; reviewed in [10, 11, 13]) and benefits have never been shown in randomized trials. For decades the management of brain AVMs has been based on a comparison between the risks of conservative management, or the so-called natural history of the disease (the risks of rupture according to various characteristics of a particular lesion in a particular patient) and the risks of treatments judged to be most appropriate for each patient. Although this rationale makes intuitive sense, it is based on assumptions that have never been validated and on extrapolations of data from error-prone observational studies over the lifetime of patients with AVMs [25].

The natural history studies indicate an overall risk of initial hemorrhage of approximately 1–4 % per year, although the long-term consequences in terms of the probability of death or long-term disability following ICH remain unclear [26–31]. Mortality from the first hemorrhage has been reported to occur between 10–30 % of patients with a ruptured AVM, but some recent data suggest that the mortality rate may be lower, and perhaps only 10–20 % of survivors have long-term disability [27, 30]. Hemorrhagic presentation is considered the most reliable risk factor for a repeat hemorrhage [26, 27, 29]. Other risk factors, such as deep venous drainage, are less consistently identified, and typically may entail not only a higher risk of rupture without treatment, but also a higher risk of treatment. Thus, the first distinction to be made regarding AVM patients is whether or not they present with ICH. Because ruptured brain AVMs presumably have a higher hemorrhage risk (4.5–34 %) than unruptured ones (0.9 –8 %) [32], interventional treatment is perhaps advisable [2, 17, 33] at least in some patients, despite the absence of evidence from randomized controlled trials (RCTs) that the benefits outweigh the risks [6]. More controversial is the management of unruptured brain AVMs.

Recently, the results of A Randomized Trial of Unruptured Brain AVMs (ARUBA), the only RCT on AVMs, were published [34]. This ambitious trial sought to compare the outcomes of patients with unruptured AVMs following random allocation of treatment versus conservative management strategies. Patients were included only if they had no prior history or imaging evidence of a previous hemorrhage. The original study plan was to randomize 800 patients and examine the primary outcome, a composite measure of stroke and death (from any cause). The design was later revised to accommodate low recruitment rates [34]. Interventional management, when allocated, was decided locally by the care team and included endovascular, surgical, and/or radiation therapy, alone or in combination. The study was stopped by the Data Safety Monitoring Board (DSMB) after 6 years, and after enrollment of 223 patients who had been followed a mean of 33 months. The primary outcome was observed in 11 patients randomized to medical management (10 %) and 33 patients randomized to interventional therapy (29 %). Whether or not this disparity in outcome will persist as the study continues remains to be seen. The results confirm previous data with a 2.2 % (95 % CI 0 · 9–4 · 5) annualized hemorrhage risk for patients followed up without interventional therapy. Although ARUBA does offer the most reliable information on unruptured AVMs so far, the study has been criticized in many ways [35–38]: recruitment was meagre, with approximately one patient per year per center; generalizability of results has been questioned, given the small total number of patients and the small proportion of screened patients that were actually included in the study (226/1740 or 13 %) [25]. Only five patients were allocated to neurosurgery alone (12 combined with embolization), even though 76 (65 %) of the patients in the treated group were Spetzler-Martin (SM) grade I or II, which are cases typically managed with surgical resection alone, the modality considered to be the “gold standard” for these lesions in many centers, particularly in North America. For a disease with a long natural history, a mean follow-up of 33 months (SD 19 · 7) has been considered too short to support the potential benefits of curative treatments or to meaningfully compare events. The short follow-up favours the medical management group because all risk is accepted early with interventional therapy. Finally, the primary endpoint of stroke or death, with “stroke” defined as “any new focal neurologic deficit, seizure, or new onset headache associated with imaging findings of hemorrhage or infarction” may have included too many minor or transient events, considering that curative treatment aims to reduce lifetime risks of life-threatening or debilitating hemorrhages [36]. Because of the small total number of patients, heterogeneity of the population, and non-standardized management choices, the role for curative treatments of unruptured brain AVMs may remain uncertain, even once the long-term results of ARUBA become available. In the meantime, in the presence of such uncertainty, some authors have declared invasive curative treatments to be “experimental therapy” [39]. A recent observational study of Scottish cohorts followed for 12 years, reporting statistics similar to ARUBA, concluded that conservative management of unruptured AVMs was superior [40]. Although no clinical trial data exist on the effect of interventional therapy even after AVM hemorrhage, the most contentious issue at present is whether interventional therapy should be offered to patients with unruptured AVMs. In such patients, the best management strategy remains unknown, but the burden of proof is on those offering the potentially risky preventive interventions. In such a context, interventions may be best proposed in the context of a care trial [41]. TOBAS (Treatment of Brain Arteriovenous Malformations Study) is a study that addresses this crucial question of conservative versus interventional management for AVMs judged appropriate for either management paradigm.

The next important problem addressed by TOBAS is the role of adjunct embolization in patients with ruptured or unruptured AVMs treated with surgery or radiosurgery. Although endovascular AVM embolization can occasionally eradicate lesions before surgery or radiation therapy [20], and although embolization may potentially improve the safety and efficacy of surgical or radiosurgical treatments, it remains contentious whether it is worthwhile to accept the additional risks of endovascular treatment for a greater overall benefit for patients with brain AVMs that are treatable by surgery or radiation therapy alone. It is possible that the overall morbidity and mortality of the combined interventional management strategy is increased when embolization is added to a surgical or radiosurgical procedure [24, 42]. Therefore, pre-surgical or pre-radiosurgical embolization can be offered, but given such uncertainty, it may be best offered only as a randomized allocation between embolization or no embolization, within the context of a proper trial. This second randomized allocation has, therefore, been nested within TOBAS.

### Objectives

The general objective of the TOBAS trial is to offer a care trial context for the management of patients with brain AVMs (ruptured or unruptured) [41].

The primary objective of the first randomized study is to compare the effect of conservative versus interventional management (i.e. neurosurgery, radiosurgery, embolization, alone or combined) on a composite of disabling stroke or death from any cause at 10 years in patients with unruptured AVMs (patients with ruptured AVMs will be analyzed separately in secondary analyses).

The primary objective of the second randomized study is to compare the effects of embolization prior to neurosurgery or radiotherapy versus neurosurgery or radiotherapy alone, in the management of patients with ruptured or unruptured AVMs, on a composite outcome of complete obliteration of the AVM combined with an independent functional outcome at the end of the management plan.

## Methods

### Trial design

TOBAS is a prospective study that includes 2 multicenter pragmatic 2-arm parallel group RCTs using a 1:1 ratio and a clinical registry of patients managed outside the RCTs. The complete protocol is available at www.clinical-care-trials.org.

### Patients

All patients with an AVM diagnosed at a participating clinical center will be candidates for the study. Patients with unruptured AVMs considered for curative treatment, without contraindication to intervention, will be candidates for the randomized allocation of management between interventional and conservative management. All patients judged by the multidisciplinary team as potential beneficiaries from endovascular therapy as pre-surgical or pre-radiosurgical treatment will also be candidates for the nested randomized study on pre-embolization. The nested study is a randomization between embolization or no embolization for patients allocated to or being prescribed surgery or radiation therapy (when treatment is judged possible with or without embolization). Patients may be referred for enrollment by their clinical neurologist, neurosurgeon, or interventional radiologist.

### Participating centers

Participating centers are expert referral centers that can provide multidisciplinary and multimodality care for brain AVMs. There is no requirement for a minimal number of procedures (per center or per surgeon per year) but the experience of centers will be recorded.

### Eligibility criteria

TOBAS is a pragmatic care trial [41]. Any patient with a brain AVM can be included in the randomized trial or registry portion of the study.

### Study interventions

All patients participating in the trial will receive “standard medical care,” including stroke risk factor reduction (smoking, hypertension, diabetes, hyperlipidemia) as routinely, locally performed, irrespective of treatment allocation.

### Medical management

Patients allocated to the medical management arm will receive no additional interventional treatment.

### Interventional therapy

A patient allocated to interventional therapy is expected to begin interventional therapy within 3 months following randomization. Interventional therapy consists of either endovascular attempts to occlude the nidus and feeding vessels, microsurgery for resecting the AVM itself, or radiosurgery, alone or in combination, and with the various locally recommended timing of each treatment session. Interventions are applied flexibly, as in normal practice, and there is no standardization required per protocol.

Endovascular treatment may include AVM embolization, or even coiling of aneurysms in the vascular territories feeding the AVM (AVM-related aneurysm). The embolization materials used for those who undergo embolization as part of the treatment plan will be limited to those agents approved by the Food and Drug Administration (FDA) or by the approval agency applicable to the country in which the patient receives treatment at the time of the procedure. This plan allows for the introduction of new agents during the course of the study. The name of the agent and the frequency of use will be recorded on the Interventional Therapy Form.

Microsurgery may include AVM resection, and aneurysm clipping related to AVM. The operating time and the necessity of transfusions will be recorded on the Interventional Therapy Form.

Radiotherapy involves the targeting of the AVM nidus and adjacent vessels intended to induce a reduction in AVM size, and possible obliteration, of the AVM. Based on local patterns of practice, variations exist in the type of equipment used, the methods of measurement used to assess the location and size of the AVM chosen for therapy, the individual doses and numbers of treatments, and whether radiosurgery is used before or after embolization or microsurgery. The modality, energy, number of isocenters, collimator size, prescription and duration of treatment will be recorded on the Interventional Therapy Form.

The goal of the interventional therapy is to achieve eradication of the AVM. The eradication plan may include any or a combination of endovascular, surgical, or radiotherapy treatments. Following interventional therapy, using a diagnostically relevant imaging study, treatment outcome will be documented as: complete AVM removal or occlusion, or incomplete AVM removal or occlusion.

### Outcomes

The primary outcome of the first RCT and of the registry is a composite of disabling stroke (resulting in modified Rankin Scale (mRS) > 2) or death from any cause at 10 years. Functional outcome status will be measured by the modified Rankin Scale, the most widely used outcome measure for stroke [43], using a standardized questionnaire administered by unblinded care personnel.

Secondary outcomes include overall mortality (all causes), overall morbidity (mRS > 2; all causes) at 1, 5 and 10 years, the occurrence of any neurological event during follow-up, the incidence of permanent (more than 3 months) disabling (mRS > 2) peri-operative (within 31 days) complications, the incidence of any peri-operative (within 31 days) complication, the incidence and number of hospital admissions, peri-treatment hospitalization lasting more than 15 days, discharge to a location other than home, occurrence of an ICH after enrollment and for up to 10 years, and occurrence of failure to eradicate the AVM (as determined by angiography) using the intended treatment modality.

The primary outcome of the nested RCT on pre-surgical or pre-radiation embolization is complete obliteration of the AVM (judged by local angiographers) combined with an independent functional outcome (mRS < 3; using a standardized questionnaire administered by unblinded care personnel) at the end of the management plan (no less than 3 months after the last procedure; no more than 3 years after radiation therapy).

### Number of patients

The number of patients to be recruited depends on the specific trial hypotheses:

i) Randomized comparison of interventional treatment and conservative management:

Interventional management of unruptured brain AVMs suitable to both conservative and curative treatment alternatives will lead to a decrease in the number of poor outcomes (defined as mRS >2) from 25 to 15 % at 10 years.

i) Nested trial on the role of embolization in the treatment of brain AVMs:

Pre-surgical or pre-radiosurgery embolization of cerebral AVMs can decrease the number of treatment failures (failure to achieve: (angiographic cure with good outcome)) from 20 % to 10 %.

ii) Secondary hypotheses:
  Curative treatment of cerebral AVMs can be accomplished with an acceptable up-front risk, defined as the occurrence of a treatment-related permanent disabling neurological complication in 10 to 20 % of patients or less (depending on AVM grade).
  Embolization of cerebral AVMs treatable by surgery or radiosurgery alone can be accomplished with an acceptable risk, defined as permanent disabling neurological complications of 8 % (3.4 to 12.6 %, 95 % CI).

Approximately 540 randomized patients (270 per group) are necessary to detect a hypothesized reduction of 10 % (from 25 to 15 %) in poor outcomes at 10 years, between interventional and conservative groups (all modalities included). It is too early to estimate the number of cross-overs that will occur; with a long follow-up period, losses can be substantial; analyses using the intention-to-treat principle would require a larger number, according to the proportion of cross-overs and losses to follow-up.

In theory, each management strategy should be separately validated, which would mean that for each modality, we would eventually need to recruit 540 patients or more.

For the nested study on pre-therapeutic embolization, 440 patients (220 per group) will be required to detect the hypothesized 10 % increase (from 80 to 90 %) in rate of success, defined as complete AVM eradication without the occurrence of a disabling complication.

### Randomization and allocation of treatment options

**Fig. 1:**
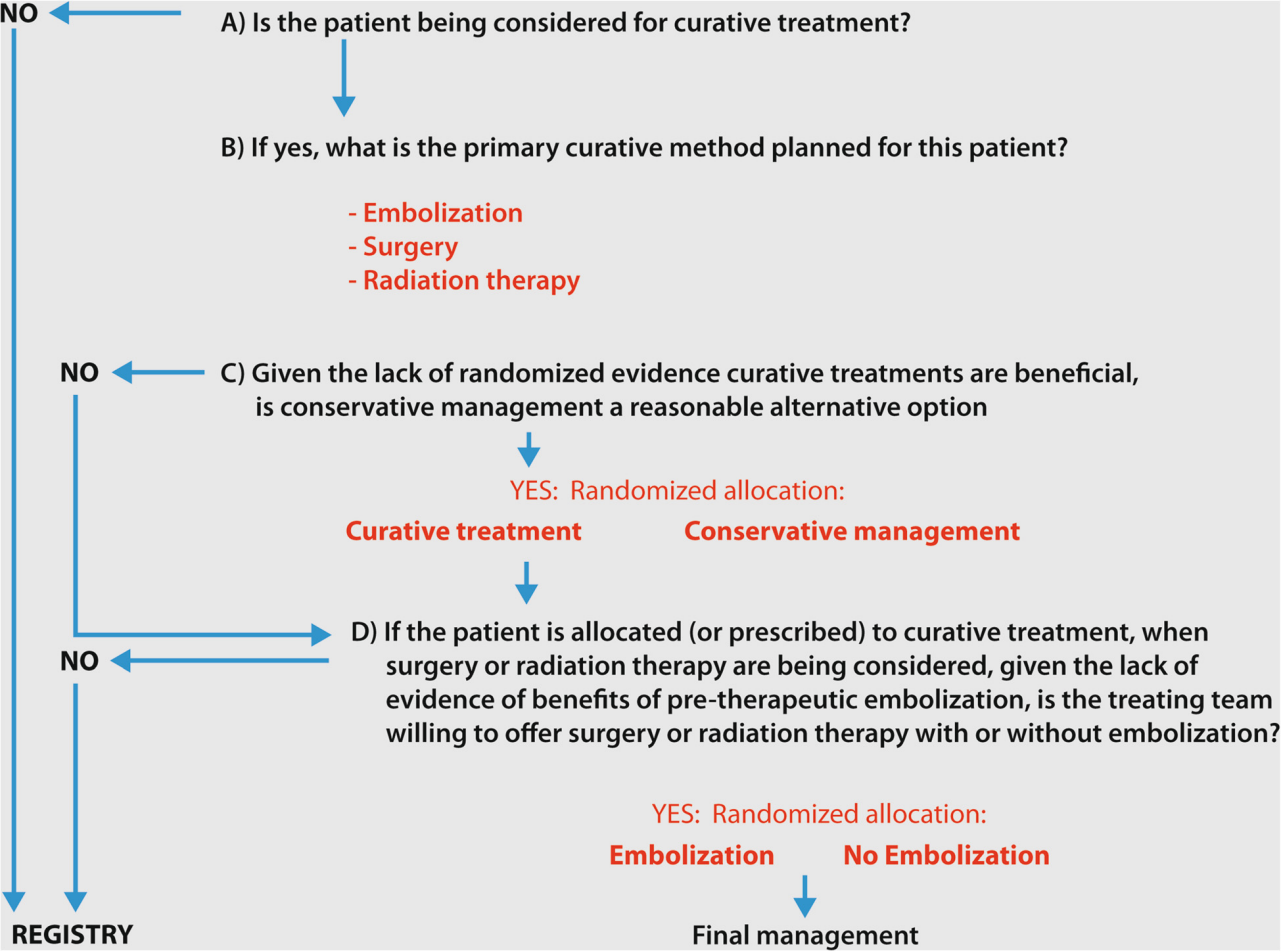
Treatment allocation. The figure illustrates the list of questions (**a**-**d**) used by the multidisciplinary team to allocate treatments by clinical judgment (**a**-**b**) and/or randomization (**c**-**d**)

Patients will be allocated a management option according to a combination of clinical judgment and randomization, as shown in Fig. 1. Patients for whom interventional management is not indicated will be proposed for conservative management and entered in the registry. Patients presenting with unruptured AVMs (and no other formal indication or contraindication for therapy) and considered for definitive therapy will be allocated interventional treatment or conservative management by randomization (ratio 1:1). The treatment modality to be used in the case of interventional management will be predefined according to clinical judgment prior to randomized allocation stratified according to treatment modality.

Some patients with difficult but ruptured AVMs, or patients that have presented with rupture in the past, but who remain untreated, may also be offered randomized allocation if the treating team judges that they are eligible for both management options.

The interventional therapy arm of the trial involves a plan for eradication of the AVM using: 1) surgical resection when the lesion is considered by a multidisciplinary team to be safely “operable,” or; 2) radiation therapy when the AVM is smaller than 3 cm, and considered to not be safely “operable,” or; 3) curative embolization, when the lesion is considered curable by embolization (with or without adjunct treatments when embolization turns out to be incomplete). Any treatment modality, if judged by the team to be appropriate, can potentially be combined with endovascular embolization.

Patients with AVMs that the multidisciplinary team expects might benefit from endovascular treatment prior to surgical resection or radiation therapy will be pre-randomly allocated to embolization or to no embolization, provided that treatment without adjunct embolization is also felt to be possible.

The patient is registered via a web-based system (http://www.medscinet.com/tobas) that will structure the forgoing allocation process and immediately communicate the result of the randomization (when appropriate) to the multidisciplinary team. The web-based platform is available 24 hours a day and assures concealment of the sequence until interventions are assigned. The first randomization will be stratified according to intended primary interventional management groups (surgery, radiation therapy, or embolization). A minimization algorithm will be used to ensure balance between groups with respect to: 1) presentation (hemorrhagic (Group I) versus all other presentations (Group II)); 2) according to SM AVM grade (I–II versus III–V). After the patient has been pre-randomized and placed in the appropriate arm of the study, the treatment team will verify that the intended management course is appropriate and considered optimal care for the patient. The patient will then be presented the recommendations of the multidisciplinary team (including the allocation by randomization when appropriate) and will be offered participation. There will be full disclosure of the pre-randomized nature of the study (Zelen’s design [44, 45]) to the patients, when this procedure will be used. They will then be asked to sign the consent form. Patients not willing to comply with the proposed allocation will still be offered participation in the study, but a cross-over will be recorded. Patients treated according to clinical judgment alone will be entered in the registry portion of the study (after the consent form is signed).

Justification of stratification, minimization criteria and other design choices such as Zelen’s are provided in Additional file 1.

### Planned patient follow-up and data collection

All patients will be seen in clinic 6–8 weeks after treatments, at 6 months, 1 year, and yearly thereafter, as part of routine follow-up care. These intervals will serve to determine mRS scores, and to inquire about possible neurological events and hospital admissions. Patients treated by embolization or surgery are typically followed up with catheter angiography 3 months after the last treatment session, to prove definite eradication of the lesion. Patients treated by radiosurgery are typically followed up by yearly magnetic resonance angiogram (MRA) and catheter angiography 2–3 years after irradiation. Patients managed conservatively are typically followed-up by MRA at yearly intervals, with the interval to non-invasive imaging increasing with time and demonstrated stability. Thus all patients will have invasive or non-invasive imaging (computed tomography angiogram (CTA) or MRA) 3 months to 3 years post-treatment (depending on treatment modality) to determine the presence of a residual nidus. This follow-up is considered to be standard of care. A minimal follow-up imaging program, in treated or untreated patients, should include MRI and MRA at 1, 5 and 10 years. We have minimized the number of tests and visits for patients. The case report forms (CRFs) can be filled at the time of standard clinical visits. There is no extra test, risk or cost beyond what is considered normal care (Table 1).

**Table 1:** Schedule of evaluation

Evaluation	Screening	Pre-entry	Entry	Treatment	Discharge	6 months and yearly	10 years
Documentation of AVM	X						
Imaging^a^	X					X	X
Informed consent			X				
Medical treatment/history		X					
Clinical assessment		X				X	X
Neurological exam		X				X	X
Failure to occlude AVM				X			
Number of days in hospital					X		
Discharge disposition					X		
Residual nidus						X	X
Hemorrhage during FU						X	X

^a^Including catheter or non-invasive angiography and brain magnetic resonance imaging (MRI) imaging whenever clinically indicated

*AVM* arteriovenous malformation, *FU* follow-up

### Statistics

There are many components to this study (Table 2). It is important to distinguish the two randomized levels, which will be analyzed separately as randomized trials (intention-to-treat and per-protocol), and the registry portion, analyzed as an observational study.

**Table 2:** Predefined group and subgroup analyses according to treatment allocation

Stratified randomized allocation	Groups: Intervention group versus Observation group
	Surgery subgroup versus Observation subgroup
	Radiosurgery subgroup versus Observation subgroup
	Embolization subgroup versus Observation subgroup
Randomization on pre-embolization	Groups: Embolization versus No embolization
	Embo + surgery subgroup versus Surgery alone subgroup
	Embo + radiosurgery subgroup versus Radiosurgery subgroup
Registry	
	Conservative management
	Surgical treatment (alone)
	Surgical treatment (with embolization)
	Radiotherapy (alone)
	Radiotherapy (with embolization)
	Embolization (alone)
	Embolization + surgery
	Embolization + radiotherapy
According to presentation	Unruptured AVM patients
	Ruptured AVM patients
According to SM grading	Low-grade AVM patients
	High-grade AVM patients

*AVM* arteriovenous malformation, *SM* Spetzler-Martin

Descriptive tables on demographic variables and potential risk factors will be provided to describe the groups at baseline. Continuous variables will be summarized in tables and will include the number of subjects, means, standard deviations, medians, minima and maxima. Categorical variables will be presented in tables as frequencies and percentages.

For the first RCT (interventional or conservative management strategy), the primary analysis, concerning the primary outcome (mRS > 2 or death from any cause at 10 years) of unruptured AVM patients will be analyzed using a Fisher’s exact test and the odds ratio with 95 % confidence interval will be reported. The same analysis will be performed for the ruptured group (considered a secondary analysis). All other secondary objectives and analyses for this first RCT will be analyzed in the same way.

The primary outcome of the second RCT (complete obliteration of the AVM combined with a mRS < 3; pre-surgical or pre-radiation embolization versus no embolization) will be analyzed with a logistic regression adjusted for ruptured status and intervention. Adjusted odds ratio and 95 % confidence interval will be reported. Moreover, stratified results by ruptured status and intervention will be presented.

The patients entered in the registry will be included in secondary analyses designed to estimate treatment morbidity for each modality and natural history of untreated patients, just as an observational study, for unruptured and ruptured AVMs, for high-grade and low-grade lesions. There are four groups and eight registered subgroups (Table II). Predetermined subgroups that will be examined include: 1) according to treatment modality; 2) according to presentation (hemorrhagic versus all other presentations) and 3) according to SM AVM grade (I–II versus III–V). With a 10-year follow-up, it is likely that there will be losses to follow-up, but it is too early to estimate how many. Primary analyses will include three categories (good outcome; bad outcome; lost to follow-up). We will explore results when a best case scenario (patients lost to follow-up have a good outcome) and a worst case scenario (they have a bad outcome) are imputed.

All analyses will be done with SAS software (SAS Institute Inc., Cary, NC, USA) using a significance level of 5 %.

### Trial monitoring

Monitoring of trial data quality will be web-based and performed by periodic reviews of data stored in the database. Blinded data will be prepared for periodic safety reviews at pre-specified intervals by an independent Data Safety and Monitoring Committee (DSMC) to ensure patient safety. Serious adverse events will be tabulated for the registry and randomized trial sections of the study, separate or aggregated, a) per management group (interventional or conservative), b) per treatment group (observation, surgery, embolization, radiation therapy) and c) according to minimization criteria (ruptured or unruptured; SM I–II or more). More specifically, the DSMC will ensure that treatment-related complications are within the confidence intervals compatible with the study hypotheses. The DSMC will also monitor the number of cross-overs as the study progress, to adjust the hypotheses or change the design. The DSMC will be composed of benevolent physicians (at least one neurosurgeon, one radiologist, one neurologist) not involved in the conduct of the trial, as well as a statistician and an ethicist. A DSMC charter predefined all trial monitoring procedures. Unblinding criteria will be pre-specified in the DSMC charter.

### Ethics approval

The Institutional Review Board (CER) of the *Centre Hospitalier de l’Université de Montréal* (CHUM) approved the protocol on 22 October 2013 (Study ID: 13.315). Secondary approval will be obtained from all local ethics committees. Recruitment will not begin in any individual center until all local ethical approvals have been obtained. Based on the Declaration of Helsinki, written informed consent will be obtained from each participating patient or appropriate surrogate in oral and written form prior to enrollment.

## Discussion

One of the difficulties that has delayed the design of trials on AVMs is the relatively small number of heterogeneous patients treated by multiple treatment modalities, which renders well-powered randomized studies addressing specific questions on each treatment option difficult to conceive. To mitigate this difficulty, we have chosen to design a single trial and registry and keep inclusion criteria as broad as possible. The reason is that the most urgent necessity is to provide patients with a randomized trial context within which promising but unvalidated care can be provided in a large collaborative effort. This choice of study design has two correlates: the first is that as currently conceived TOBAS raises a general question as to whether unruptured AVMs should be treated. Although the number of patients for whom the uncertainty is present may be quite large, reasons are multiple and varied. For many clinicians these are as many, distinct research questions regarding what are considered heterogeneous groups of patients or lesions that should not be lumped together into an overall outcome result, for fear of averaging results that would have pulled in opposite directions. We must, however, remember the first goal of care trials: to protect present patients from unjustified beliefs and hypotheses, and offer optimal care in the presence of uncertainty [41]. From an organizational perspective, it is easier to propose a single inclusive large trial than to multiply small trials with narrow selection criteria. TOBAS could be split into five different trials or more. Given the difficulties with recruitment that were previously encountered in ARUBA, it is difficult to predict which ‘trial’ would recruit. It is possible, however, to pre-specify subgroups that will be individually monitored by the DSMC. Thus, the second implication of our design choice is that the DSMC must regularly be provided with subgroup-specific safety data to ensure that an emerging obvious discrepancy in clinical outcome will be addressed in a timely manner in order to prevent additional patient morbidity. We will then be in a position to identify which treatment modality and which patients should be submitted to randomized allocation as the trial progresses. Because each treatment modality should eventually be validated as beneficial before it becomes accepted as standard care, we may have to adjust hypotheses and the number of recruited patients as the trial proceeds, to eventually come up with answers that can apply in clinical practice.

We have previously been confronted with the difficulties involved in recruiting patients in trials comparing invasive treatments and conservative management [46]). In this particular case, difficulties are multiplied by the number of possible treatment modalities, in isolation or combined, and by the fact that not all options are possible for a particular patient. Thus, allocation of treatments is a combination of clinical judgment and randomization. We believe pre-randomization is particularly suited to this context [44, 45]. For each patient, it will clearly be explained which portion of the eventually recommended treatment plan was decided using the clinical judgement of the expert team, and which part of the treatment plan was randomly allocated.

The neurovascular community has recently voiced discontent regarding the way clinical trials have been designed and conducted in the past decade [47, 48]. While some criticisms may be unfair, the general feeling is that trials are slow, rigid, costly, heavy duty machineries that eventually recruit only a small proportion of patients being treated with the target interventions, eventually providing results that cannot apply to clinical practice. We must proceed with fundamentally different sorts of trials [49].

Physicians involved in the treatment of brain AVMs believe that interventional treatments are indicated to prevent the lifetime risks of rupture or re-rupture and resulting poor outcomes. Unfortunately, the benefits of treatment have never been proven in a RCT, and any treatment entails immediate risks. Although all treatment modalities have been performed in expert centers for decades, it is difficult to estimate treatment-related risks for a particular patient because they vary tremendously from one patient to the other, from one modality to the other, and perhaps according to local expertise, making clinical results published in the medical literature strongly dependent on case selection and comparisons between case series unreliable. What we can do with the current non-randomized observational studies is estimate the treatment risks and chances of success of this sort of patient compared to this other sort of patients, but this reasoning should not make the treatment of a patient justified any more than observation of the other. If our experience and clinical intuition suggest that individualized clinical decisions can be based on a theoretical comparison between the supposed lifetime risks of hemorrhage, and treatment-related risks (an unreliable process at best), the resulting singularized practice can never be validated as beneficial. In effect, acting on an individual basis, examining the results after the fact, can never tell us what would have happened had the other management option been chosen. This approach blocks any subsequent attempts to demonstrate the benefits of curative treatments for any group of patients. When a trial is finally proposed, the current clinical culture foists upon us the idea that we are sacrificing the care of individuals for the sake of gaining scientific knowledge, and both patients and physicians become reluctant to enroll [46, 47]. For a practice to be validated as beneficial, we need similar patients that can be managed by two management options, and we need to show results that are better with one treatment than the other, when they are randomly allocated.

The main differences between the present study and the ARUBA trial are: a) a primary hypothesis in favor of interventions; b) a harder primary endpoint; c) a longer follow-up period; d) all inclusive selection criteria. The hypothesis of the ARUBA trial was that conservative management of unruptured AVMs was superior to interventional management. No evidence is necessary to support the hypothesis that conservative management is less risky in the short term. While the ARUBA results are a reminder that by attempting preventive treatments of brain AVMs we may be doing more harm than good, should all attempts to cure unruptured brain AVMs now be stopped? In fact, the burden of the proof was not on neurologists who believed unruptured AVM patients should be left alone; it has always been on the physicians proposing those promising but risky preventive interventions that have yet to be validated as beneficial. Unvalidated risky preventive treatments should not be prescribed just as validated care. The current context of brain AVM therapy calls for a different approach.

Care trials have been designed to offer optimal care in the presence of such uncertainty. If validated care can simply be prescribed and acted upon, unvalidated care can be offered, but only as a 50 % chance of receiving the target intervention, and a 50 % chance of receiving the validated alternative (or conservative management when none exists), until the uncertainty is lifted and the best management option identified. In this manner, well-intended hypotheses, such as the one underlying all our interventional practices that immediate risks are worth taking to prevent poor outcomes from future ruptures, can be transformed into better patient outcomes in real life (when the hypothesis is verified), or harmful treatments are abandoned before they do too much damage. Once a treatment is validated (but not before), it can be adopted as normal care. Self-disciplining our actions in this manner would ensure continuous progress, not only in knowledge about the disease or its treatment, but in ever improving results of our clinical actions and outcomes for our patients. If we understand this way of controlling unvalidated risky preventive clinical interventions, we understand that proposing randomized allocation of treatment options is not performed for the sake of gaining knowledge. In this context it is the way to offer optimal medical care, until the uncertainty is lifted by the very trial being offered, and the best option is identified.

We have chosen a relatively hard primary endpoint (death and dependency) observed after a relatively long follow-up period (10 years), because in most circumstances, physicians and patients are willing to accept substantial risk to eradicate a lifetime AVM hemorrhage risk. Furthermore, it is ill-advised to abandon curative treatments due to transient or minor complications, especially when it is possible that the follow-up period has been too short to identify the risks of conservative management. This means that a large number of patients followed for a long period will be needed before definitive conclusions can be drawn. In our view, this is appropriate if we keep in mind the aim of the study. We hope physicians already dedicating their professional lives to offer highly specialized costly treatments for these difficult patients will understand that although everyone would like to have results as soon as possible, more expedient studies using surrogate endpoints or short follow-ups can only provide risky premature conclusions. Adjustments in trial design are needed, however, such as minimal intrusion in patient care, simple CRFs that can be filled by care personnel, and no extra test or cost, to ensure that results are not biased by the necessity to control trial duration or expenses (when conventional trials necessitate high monetary compensations) and that for the entire duration of the trial participation is meant to be optimal care in the presence of uncertainty. Secondary endpoints were chosen to allow the DSMC to monitor immediate treatment results and ensure that harmful interventions are identified and stopped as early as possible.

The same reasoning applies for the nested trial on pre-surgical or pre-radiosurgical embolization. Although many surgeons strongly believe embolization is helpful in ensuring a safer and more complete surgical resection in many cases, this benefit has never been proven. The benefits in the operating room may end up being negated by the up-front risks of embolization. The same considerations are involved in trying to improve the efficacy of radiosurgery for AVMs that are considered too large to be treated by this treatment modality.

None of the AVM treatment options have been validated so far, for any group of patients. This is to justify the all-inclusive study design. It is possible to propose random allocation even to those patients with ruptured AVMs when risks of treatment are felt to be high (perhaps as high as risks of not treating them), or when complete eradication may be difficult. On the other hand we understand that some beliefs are so entrenched that they may have become impossible to question [46]. This is the rationale for including the registry. For some patients with unruptured AVMs that are felt, by some collaborators, to be so safely curable that treatment is considered mandatory; they can be part of the registry. For example, if surgical resection of SM grade I patients is shown to be very safe in multiple centers (say with M&M less than 5 %, with narrow confidence intervals), perhaps this treatment modality may be justified without randomized evidence for that subgroup of patients. The registry will also permit the identification of the patients who were treated outside the randomized part of the trial, and help in the interpretation of the generalizability of trial results, a common concern with previous trials in the neurovascular field [47]. The registry is also meant to encourage the recruitment of as many expert centers as possible, so that all can collaborate in trying to offer optimal care for this difficult medical condition. We hope to convince all participants that randomized allocation is best for each individual, but it may take much more time before this notion becomes widely accepted by the clinical community. Although the registry data may be difficult to interpret, it may provide valuable insight into the various treatment modalities, particularly for ruptured AVM patients. The current practices where AVMs are treated on a case-by-case basis leave much to be desired. Patients are better served by being treated within a care research context, where, instead of having a 100 % chance of being allocated an unvalidated invasive treatment, they are offered a 50 % chance of getting a potentially beneficial treatment, and an equal 50 % chance of escaping the risks of a potentially deleterious intervention. In the spirit of care trials, the protection of current patients must always be given first consideration. We thus accept that scientific hypotheses and the number of patients required to show significant differences may need to be modified as the trial progresses.

Finally, although TOBAS was designed to include all patients, it cannot serve to answer all questions. For example, it was not conceived to provide valid comparisons between interventions, for patients eligible for two or three various interventional options (surgery versus radiosurgery; surgery versus embolization; embolization versus radiosurgery).

Treatment of brain AVMs has never been validated as beneficial. In the presence of such uncertainty, optimal care has to be provided in a special context combining research and care. TOBAS provides such a care trial context to help the collaborative efforts of multiple expert centers in providing optimal management of patients with brain AVMs immediately and eventually to lift some of the uncertainty regarding best management options.

## Trial status

TOBAS is currently recruiting patients in one Canadian center, and the protocol is under ethics board review at other national and international sites. To date, 80 patients have been recruited.

## Additional file

Additional file 1:
**Appendix.** (DOCX 27 kb)

## Abbreviations

ANOVA: analysis of variance
ARUBA: A Randomized Trial of Unruptured Brain AVMs
CER: Institutional Review Board
CHUM: *Centre Hospitalier de l’Université de Montréal*
CRF: case report form
CTA: computed tomography angiogram
DSMB: Data Safety Monitoring Board
DSMC: Data Safety and Monitoring Committee
FDA: Food and Drug Administration
ICH: intracranial hemorrhage
TOBAS: Treatment of Brain Arteriovenous Malformations Study
AVM: arteriovenous malformation
MRA: magnetic resonance angiogram
MRI: magnetic resonance imaging
mRS: modified Rankin Scale
NIS: Nationwide Inpatient Sample
RCT: randomized controlled trial
SD: standard deviation
SM: Spetzler-Martin grade
