# Supplementary material for: Treatment of Brain AVMs (TOBAS): study protocol for a pragmatic randomized controlled trial
Source: Trials. 2015 Nov 4;16:497. doi: 10.1186/s13063-015-1019-0 (PMC4632683; doi:10.1186/s13063-015-1019-0)
Supplement: Additional file 1: — Appendix. (DOCX 27 kb) [file 13063_2015_1019_MOESM1_ESM.docx]

**Additional file 1**

**Treatment of Brain AVMs (TOBAS): study protocol for a pragmatic randomized controlled trial**

**Minimization criteria:**

A) Haemorrhagic presentation is the most frequently recognized risk factor for (re-) haemorrhage, and is currently the most frequent indication for curative treatment of brain AVMs. Results will be separately analyzed for ruptured and unruptured AVM patients;

B) The Spetzler-Martin grading system has been validated as predictive of surgical morbidity [43]. It is also the most frequent classification system that globally describes all brain AVMs according to size, eloquence of the surrounding brain, and venous drainage, important characteristics that should be balanced between groups for the future interpretation of study results.

**Stratification:**

Stratification per intended primary interventional management group (predetermined prior to randomization) will ensure comparability of the primary endpoint for patients managed conservatively versus actively for each treatment modality. This is necessary to ensure that the control groups are appropriately matched to the experimental groups. For example, patients for whom surgery would have been an option, but did not receive surgery, will be compared with those patients who did receive surgery. Importantly, because TOBAS is an all-inclusive care trial, capable of validating the benefits of a primary treatment modality, results will be analyzed separately for each treatment modality, in order not to confound results of a potentially beneficial treatment modality with results of one that is potentially detrimental.

Because results from any trial can only cover a predetermined, relatively short follow-up phase (as compared to a life time risk of rupture), and because the treatment of cerebral AVMs cannot be accomplished without significant risks, within the secondary hypotheses we have separated the notion of clinical efficacy (such as the number of neurological, haemorrhagic events during follow-up, and angiographic cure rates according to modality), from the notion of initial ‘costs’ of treatments (in terms of risks of peri-operative morbidity and mortality) for each modality.

**Clinical judgement and (Zelen) Pre-randomization**

Because AVM patients and the lesions themselves are very heterogeneous, with some AVMs readily treatable but others very challenging, (with an accompanying range of uncertainty regarding treatment indications), all patients will be studied by a multidisciplinary team to make the best treatment recommendation (including an assessment of whether they are appropriate for randomized allocation to one, the other or both randomized studies). For patients determined appropriate for the trial, the multidisciplinary team will select which portions of the trial they consider appropriate for each individual patient (ie: is this patient a candidate for the arm of the trial comparing treatment vs conservative management; or for the portion of the trial comparing embolization prior to surgical or radiosurgical management). Patients not considered to be appropriate for any randomized portion of the trial will be included in the prospective registry.

Because of the failure to recruit many patients in the previous ARUBA trial (less than one patient per year in most centres), the present study proposes pre-randomization, with fully informed consent (including information regarding the randomized allocation of treatment options and the various treatment options available) before any treatments. Failing this, they will be proposed participation in the registry where all patients will be evaluated.
